# Supplementary material for: Direct visualization of a native Wnt in vivo reveals that a long-range Wnt gradient forms by extracellular dispersal
Source: eLife. 2018 Aug 15;7:e38325. doi: 10.7554/eLife.38325 (PMC6143344; doi:10.7554/eLife.38325)
Supplement: Supplementary file 1. [file elife-38325-supp1.docx]

|  | | | |
| --- | --- | --- | --- |
| **Promoter** | **Forward primer (5' - 3')** | **Reverse primer (5' - 3')** | **length** |
| *Pegl-20* | TTTTTGAAGTCATCCTACTAACTAA | CATTATTTCTGAAATTGAGATGTTTTAGA | 1,892 bp |
| *Plin-44* | CGCCAGCACCAAAATATCCAAG | CATCACGCTGTGTCACCTCG | 1,508 bp |
| *Peft-3* | GCACCTTTGGTCTTTTATTGTC | AGCAAAGTGTTTCCCAACTG | 607 bp |
| *egl-5 K enhancer* | ACTTGCCTTTCTACCGTAGAC | GTGAAGGGGTTTGCTTTAGTT | 447 bp |
| *Pgcy-32* | CCATGGTGTTAATACGTCAAGCA | TCTATAATACAATCGTGATCTTCG | 796 bp |
| *Pmec-7* | TAAGAGCTTTCAACACCCCGC | GTTGCTTGAAATTTGGACCCGA | 628 bp |
| *Pmyo-3* | GCTATAATAAGTTCTTGAATAAAAT | TCTAGATGGATCTAGTGGTC | 2,383 bp |
| *Pwrt-2* | TAAATTTACGGGTGTTCCCCA | CATCCGAGAAACAATTGGCAG | 1,213 bp |

**Supplemental table 1. PCR primers used to amplify transgene promoters.**
